# Identification and validation of five ferroptosis-related molecular signatures in keloids based on multiple transcriptome data analysis

**DOI:** 10.3389/fmolb.2024.1490745

**Published:** 2025-01-06

**Authors:** Zhen Sun, Yonghong Qin, Xuanfen Zhang

**Affiliations:** Department of Plastic Surgery, Second Hospital and Clinical Medical School, Lanzhou University, Lanzhou, China

**Keywords:** keloid, ferroptosis, fibrosis, immune infiltration, oxidative stress

## Abstract

**Introduction:**

Keloids are a common skin disorder characterized by excessive fibrous tissue proliferation, which can significantly impact patients’ health. Ferroptosis, a form of regulated cell death, plays a crucial role in the development of fibrosis; however, its role in the mechanisms of keloid formation remains poorly understood.

**Methods:**

This study aimed to identify key genes associated with ferroptosis in keloid formation. Data from the NCBI GEO database, including GSE145725, GSE7890, and GSE44270, were analyzed, comprising a total of 24 keloid and 17 normal skin samples. Additionally, single-cell data from GSE181316, which included 8 samples with complete expression profiles, were also evaluated. Differentially expressed genes were identified, and ferroptosis-related genes were extracted from the GeneCards database. LASSO regression was used to select key genes associated with keloids. Validation was performed using qRT-PCR and Western blot (WB) analysis on tissue samples from five keloid and five normal skin biopsies.

**Results:**

A total of 471 differentially expressed genes were identified in the GSE145725 dataset, including 225 upregulated and 246 downregulated genes. Five ferroptosis-related genes were selected through gene intersection and LASSO regression. Two of these genes were upregulated, while three were downregulated in keloid tissue. Further analysis through GSEA pathway enrichment, GSVA gene set variation, immune cell infiltration analysis, and single-cell sequencing revealed that these genes were primarily involved in the fibrotic process. The qRT-PCR and WB results confirmed the expression patterns of these genes.

**Discussion:**

This study provides novel insights into the molecular mechanisms of ferroptosis in keloid formation. The identified ferroptosis-related genes could serve as potential biomarkers or therapeutic targets for treating keloids.

## 1 Introduction

Keloid scarring, a complex fibroproliferative dermatosis, remains poorly understood in terms of its pathogenesis. It is characterized by excessive collagen deposition and abnormal fibroblast proliferation (Chike-Obi et al., 2009), predominantly affecting individuals aged 10–30 years and is especially prevalent among African, Hispanic, and Asian populations (Robles and Berg, 2007). Common sites of occurrence include the chest, earlobes, shoulders, and back. Beyond cosmetic disfigurement, keloids can significantly impact mental health and restrict local limb functionality. Despite the availability of various treatments, none have emerged as definitively effective (Ojeh et al., 2020). This underscores the urgent need for deeper insights into the molecular mechanisms driving keloid pathogenesis to improve therapeutic strategies.

Ferroptosis, according to Dixon, is a type of iron-dependent cell death characterized by lipid peroxidation and iron buildup (Dixon et al., 2012). Numerous diseases have been linked to pathophysiology of ferroptosis, including neoplasms, neurological diseases, ischemia-reperfusion injuries, cardiovascular diseases, and organ fibrosis, according to recent investigations (Hu et al., 2021; Mou et al., 2019). Fibrosis is an abnormal tissue repair process often associated with chronic inflammation. Inflammatory cells, such as macrophages and lymphocytes, infiltrate the affected area and release various pro-inflammatory factors, including IL-1, IL-6, and IFN. This cascade of events further promotes the activation of fibroblasts and the deposition of extracellular matrix components. Characteristics of ferroptosis include lipid peroxidation, GPX4 inhibition, and iron accumulation, which harm parenchymal cells and lead to fibrotic lesions that abnormally collect in tissues, thereby promoting fibrosis of tissues and organs (Weiskirchen et al., 2019). Currently, the role of ferroptosis in fibrosis has been validated by multiple studies, including those focusing on renal, cardiac, hepatic, and pulmonary fibrosis. El-Horany found that in a mouse model, bleomycin-induced pulmonary fibrosis was associated with increased ferroptosis markers (El-Horany et al., 2023). Chen J et al. observed that TRIM23 inhibits hepatic stellate cell (HSC) ferroptosis by regulating p53 ubiquitination, which in turn promotes cell activation and liver fibrosis (Chen et al., 2024). Wang et al. demonstrated that modulation of the JNK/p53 pathway by the MLK3 pathway which promotes ferroptosis attenuates myocardial fibrosis (Wang et al., 2020). These studies indicate that ferroptosis plays a crucial role in regulating fibrosis, potentially promoting fibrosis in various organs and tissues.

Keloids are characterized by persistent inflammation, progressive fibrosis, and irregular patterns of cell proliferation and apoptosis (Li et al., 2022). Inflammation, excessive oxidation, and iron accumulation are all linked to ferroptosis, suggesting that this cell death process influences fibrosis via multiple mechanisms, and may play a significant role in the development of keloids. The expression and function of ferroptosis-related genes in keloids remain poorly understood. This study focuses on screening ferroptosis-related genes to investigate how ferroptosis may influence keloid formation.

**FIGURE 1:**
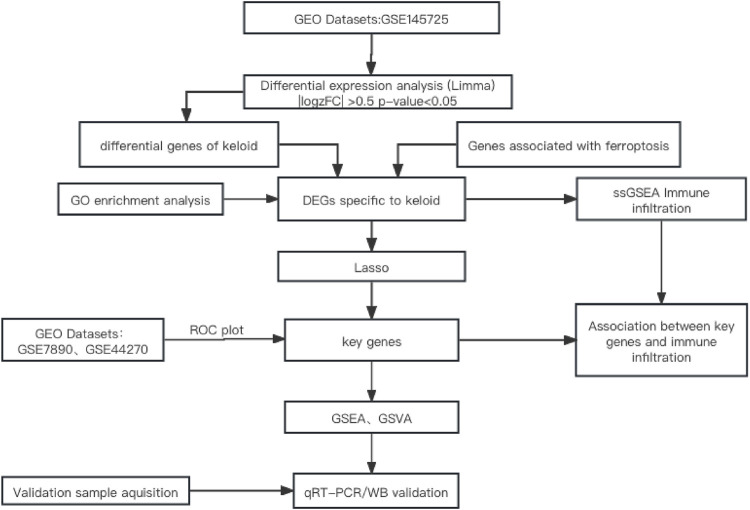
Flowchart of the research methodology.

The flowchart of the study is shown in Figure 1.

## 2 Materials and methods

### 2.1 Downloading data

The NCBI GEO public database included the Series Matrix File for GSE145725 (Kang et al., 2020), annotated as GPL16043, which contained expression profile data for 19 individuals, 10 in the normal group and 9 in the illness group. Comparably, the expression profile data for ten patients—equally divided between the normal and illness groups—were found in the Series Matrix File for GSE7890 (Smith et al., 2008), annotated as GPL570, which was also acquired from the same database. In addition, expression profile data for 12 patients—three in the normal group and nine in the illness group—were obtained from the Series Matrix File for GSE44270 (Hahn et al., 2013), labeled as GPL6244. The NCBI GEO public database included the single-cell data file for GSE181316, which contained complete expression profiles for 8 samples.

### 2.2 Analysis of differential expressions

The R package Limma is employed for differential expression analysis of expression profiles to identify genes with significantly different expression levels between groups (Ritchie et al., 2015). The “Limma” R program was used to create heat maps and differential gene volcano plots, identify genes with differential expression between disease and control samples, and analyze keloid data for variations in molecular processes. This facilitated the identification of genes with differential expression between disease and control samples. Heat maps and differential gene volcano plots were generated, with thresholds set at P Value <0.05 and |logFC| > 1 for differential gene screening.

### 2.3 Analysis of functional enrichment

The Metascape database (www.metascape.org) was used to perform functional annotation of intersecting genes to investigate their functional significance comprehensively. These genes underwent Gene Ontology (GO) pathway analysis, with statistical significance determined by a minimum overlap of ≥3 and p ≤ 0.01.

### 2.4 Predictive modeling

After selecting the potential genes, a predictive correlation model was developed using lasso regression (Friedman et al., 2010). A risk score formula was constructed for each patient based on the expression values of each gene. Weights were applied according to the predicted regression coefficients from the lasso regression analysis. This algorithm was used to compute each patient’s risk score. ROC curves assessed the model’s prediction accuracy (Robin et al., 2011).

### 2.5 Analysis of single-cell sequencing

First, the data was processed using the Seurat software, followed by the application of the t-SNE method to evaluate the data and ascertain the spatial relationships among clusters. Next, the celldex package was used to annotate the clusters, associating them with specific cells relevant to the disease’s occurrence. Finally, the FindAllMarkers function’s parameter was set to 1 to identify marker genes from single-cell expression data for each cell subtype. Marker genes were selected based on criteria of adjusted p-value <0.05 and |avg log2FC| > 1 (Tang et al., 2009; Ziegenhain et al., 2017).

### 2.6 Analysis of immune cell infiltration

Single-sample Gene Set Enrichment Analysis (ssGSEA) is a commonly used method to evaluate various types of immune cells inside the microenvironment. B cells, NK cells, and T cells are among the 29 human immune cell phenotypes that may be identified with this approach. In this study, the ssGSEA algorithm was employed to quantify immune cells within expression profiles, facilitating the estimation of relative proportions among 29 types of immune-infiltrating cells. Furthermore, a Spearman correlation analysis was performed to explore the relationship between gene expression levels and the content of immune cells.

### 2.7 Gene set enrichment analysis (GSEA)

Gene Set Enrichment Analysis (GSEA) utilizes predefined gene sets and ranks genes based on their differential expression levels between two sample classes. The analysis then determines whether these predefined gene sets are significantly enriched at either the top or bottom of the ranking list. This study employs GSEA to compare the differences in signaling pathways between high and low expression groups, thereby exploring the molecular mechanisms of key genes in both groups of patients. For the analysis, the number of permutations is set to 1,000, with phenotype as the permutation type.

### 2.8 Gene set variation analysis (GSVA)

Gene Set Variation Analysis (GSVA) is a non-parametric, unsupervised method for assessing the enrichment of gene sets in transcriptomic data (Hanzelmann et al., 2013). GSVA evaluates biological functions of samples by converting gene-level variations into pathway-level changes through an integrated scoring of the gene sets of interest. In this study, gene sets will be downloaded from the Molecular Signatures Database and the GSVA algorithm will be applied to each set to generate comprehensive scores, assessing potential biological function changes in different samples.

### 2.9 Sources of tissue samples

Ethical approval for this study was granted by the Research Ethics Committee of the Second Hospital of Lanzhou University (the project approval number is 2023A-796), and informed consent was obtained from all participants. Samples were collected from both the Plastic and Reconstructive Surgery Ward and the Outpatient Clinic at the Second Hospital of Lanzhou University. A total of five samples each of normal skin tissue and keloid tissue were collected. The exclusion criteria for keloid tissue samples included: 1) previous radiotherapy or injection therapy within the past 2 years, and 2) presence of diabetes, autoimmune diseases, or malignant tumors.

### 2.10 Quantitative real-time polymerase chain reaction and RNA extraction (RT-qPCR)

Total RNA was extracted from 5 normal tissue samples and 5 keloid tissue samples using the Trizol method. Subsequently, the extracted total RNA was reverse transcribed into complementary DNA (cDNA) using HiScript II Q RT SuperMix for qPCR (+gDNA wiper). Quantitative PCR (qPCR) was then performed using Taq Pro Universal SYBR qPCR Master Mix. The thermal cycling conditions for qPCR were set as follows: an initial denaturation step at 95°C for 30 s, followed by 40 cycles of denaturation at 95°C for 10 s, annealing at 60°C for 30 s, and extension at 60°C for 30 s. Primer sequences for each gene are listed in the attached table. The sequences of the primers for key genes are listed in (Table 1). Relative gene expression levels were quantified using the 2^−ΔΔCq method, with GAPDH as the endogenous control.

**TABLE 1:** The sequences of the primers for key genes.

Genes		Sequence	Size (bp)
HOMO_GAPDH	Forward	5′-TCAAGAAGGTGGTGAAGCAGG-3′	115 bp
	Reverse	5′-TCAAAGGTGGAGGAGTGGGT-3′	
HOMO_SOCS2	Forward	5′-GAGCCGGAGAGTCTGGTTTC-3′	239 bp
	Reverse	5′-ATCCTGGAGGACGGATGACA-3′	
HOMO_SLC38A1	Forward	5′-GCTTTGGTTAAAGAGCGGGC-3′	295 bp
	Reverse	5′-AGCTTGACACCCCTGTTAGC-3′	
HOMO_SNCA	Forward	5′-ATTCGACGACAGTGTGGTGT-3′	102 bp
	Reverse	5′-GTTTTCTCAGCAGCAGCCAC-3′	
HOMO_PLIN2	Forward	5′-TGATGGCAGGCGACATCTAC-3′	239 bp
	Reverse	5′-CTGGCTGCTCTTGTCCATCT-3′	
HOMO_AKR1C3	Forward	5′-GTCACTTCATGCCTGTCCTG-3′	249 bp
	Reverse	5′-GGACCAACTCTGGTCGATGAA-3′	

### 2.11 Western blot analysis and protein extraction

Total protein was extracted from five normal samples and five keloid samples using radioimmunoprecipitation assay (RIPA) buffer, and quantified using the bicinchoninic acid (BCA) assay. Equal amounts of protein were loaded onto SDS-PAGE. The gel was then transferred, and the membrane was blocked with 5% bovine serum albumin (BSA). The membrane was subsequently incubated with primary and secondary antibodies. The following antibodies were used: AKR1C3 rabbit polyclonal antibody, SLC38A1 rabbit polyclonal antibody, SNCA rabbit polyclonal antibody, SOCS2 rabbit polyclonal antibody (Abcam, Shanghai, China), PLIN2 rabbit polyclonal antibody, β-actin mouse monoclonal antibody, HRP-conjugated anti-rabbit secondary antibody, and HRP-conjugated anti-mouse secondary antibody (all from ProteinTech, Wuhan, China). After washing the membrane with TBST solution, the results were detected using chemiluminescence. Relative quantification was analyzed using ImageJ software for densitometry.

### 2.12 Statistical analysis

Data collection and statistical analyses were performed using R (version 4.2.2), and all tests were two-sided. GraphPad Prism (version 9.0) was used for qPCR and Western blot analyses. Data for keloid and normal skin tissues were presented as mean ± SEM. Differences between the groups were compared using Student’s t-test and Welch’s correction. A p-value of <0.05 was considered statistically significant.

## 3 Results

### 3.1 Keloid-specific DEG collection

**FIGURE 2:**
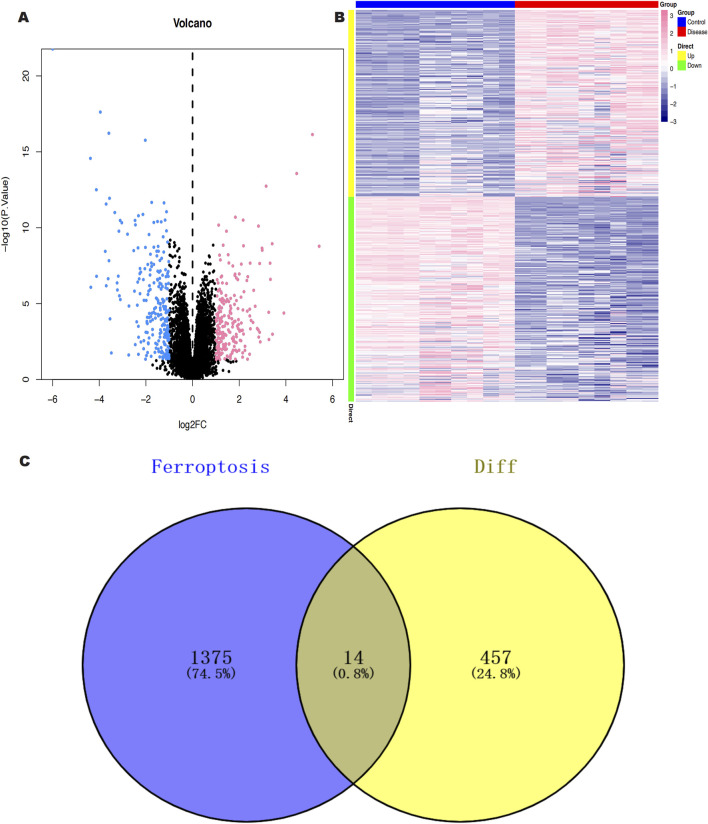
**(A)** Volcano plot of differentially expressed genes (DEGS) between keloid and normal skin samples; red color represents up-regulated genes, black color represents non-significantly different genes, and blue color represents down-regulated genes. **(B)** Heat map of DEGs between keloid and normal skin samples. **(C)** Screening of keloid DEGS intersecting with Ferroptosis genes.

**FIGURE 3:**
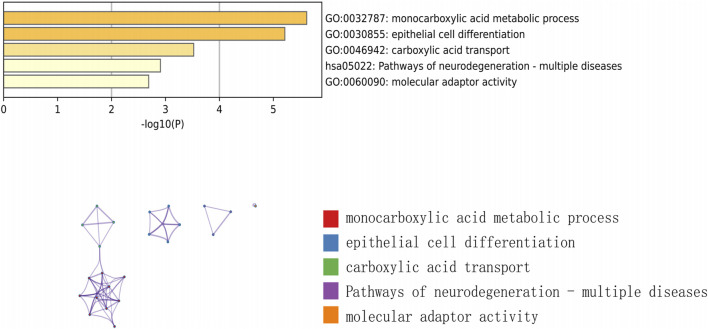
GO functional enrichment analysis of keloid ferroptosis intersecting DEGs.

We downloaded the GSE145725 dataset from the NCBI GEO public database, which included samples from 19 patients, with 10 in the control group and 9 in the disease group. Differential gene expression analysis was conducted using the limma package. Genes were selected based on the criteria of p < 0.05 and |logFC| > 1. A total of 471 differentially expressed genes were identified, comprising 225 upregulated and 246 downregulated genes (Figures 2A, B). Subsequently, we utilized the GeneCards database (https://www.genecards.org/) to extract genes related to ferroptosis with a Relevance score >1. An intersection of ferroptosis-related genes with the differentially expressed genes yielded 14 genes associated with ferroptosis (Figure 2C). Further pathway analysis on this intersected set revealed significant enrichment in pathways such as monocarboxylic acid metabolic process, epithelial cell differentiation, and molecular adaptor activity (Figure 3).

### 3.2 Construction of a predictive model for genes associated with ferroptosis in keloids

**FIGURE 4:**
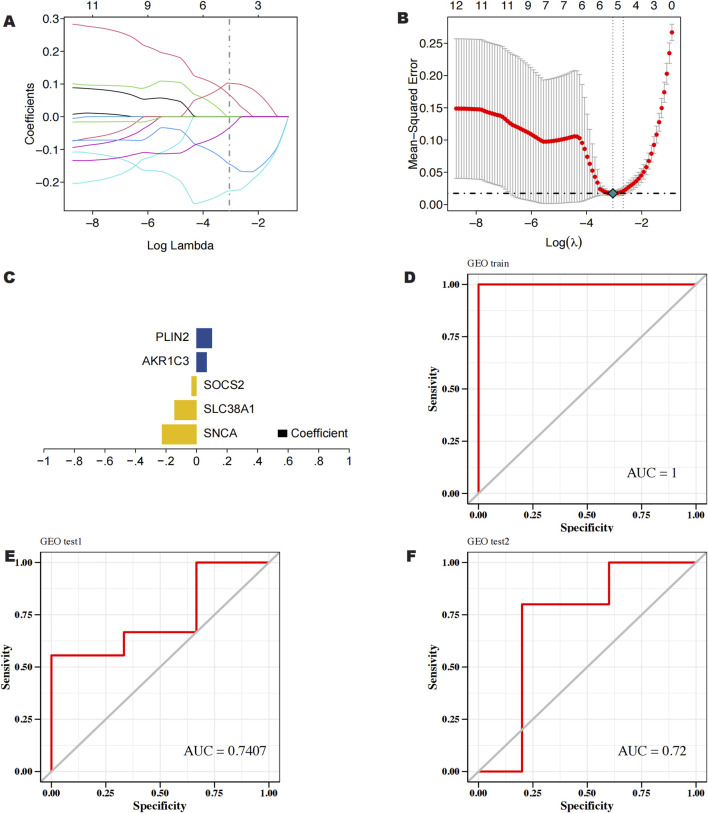
**(A, B)** Screening of characterized genes by LASSO regression analysis. **(C)** Construction of prediction models for 5 characterized genes **(D)** ROC curves of LASSO regression models for 5 characterized genes in GSE145725. **(E)** ROC curves of 5 characterized genes in GSE44270. **(F)** ROC curves of 5 characterized genes in GSE7890.

To perform feature screening by Lasso regression, we used the GSE145725 dataset as the training set and the GSE7890 and GSE44270 datasets as the validation sets. Lasso regression identified five key genes associated with keloids, which were then utilized to construct the predictive model (Figures 4A, B). RiskScore = SNCA × (−0.226932318008185) + SLC38A1 × (−0.144538726326697) + SOCS2 × (−0.033573222490279) + AKR1C3 × 0.0671905003157174 + PLIN2 × 0.102513500537292 was the model formula (Figure 4C). The prediction model (Figure 4D) showed an area under the curve (AUC) of 1, indicating high diagnostic effectiveness. AUC (Area Under the Receiver Operating Characteristic Curve) is an important performance evaluation metric, particularly suitable for imbalanced datasets, and it can comprehensively assess classification performance, ensuring the selection of the most appropriate metric to reflect the accuracy of predictive results (Parodi et al., 2022). Therefore, we employed the GSE7890 and GSE44270 datasets as external validation sets to further substantiate the model. The model’s remarkable stability was validated by the validation findings, which showed an AUC of 0.7407 (Figure 4E) for the GSE44270 dataset and 0.72 (Figure 4F) for the GSE7890 dataset.

### 3.3 Single-cell sequencing analysis

**FIGURE 5:**
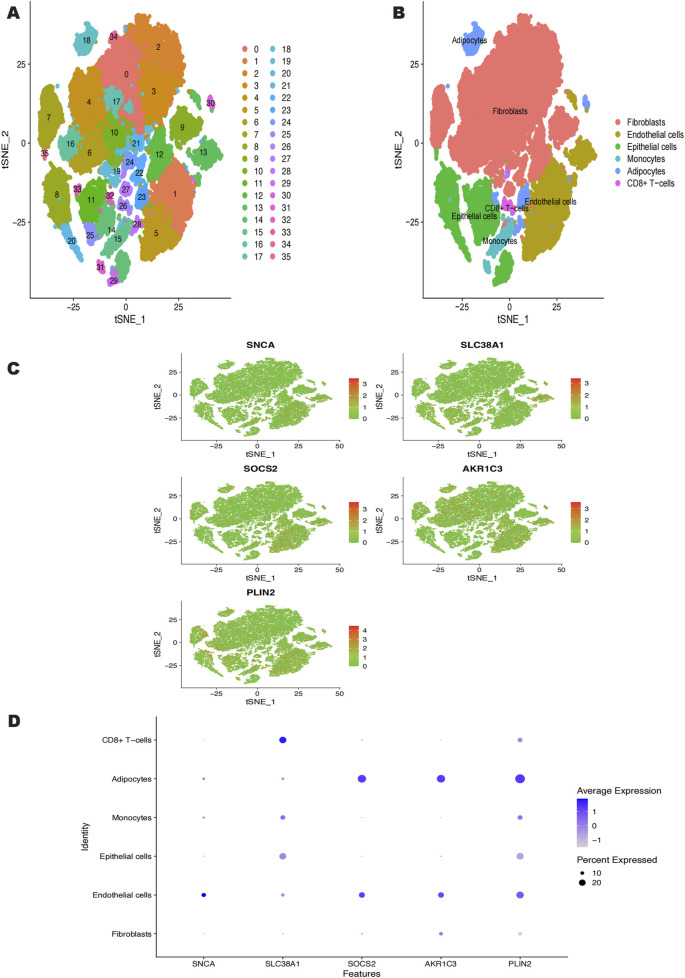
**(A)** GSE181316 single cell sequencing analysis, 36 isoforms were obtained. **(B)** 36 clusters were annotated into 6 cell categories, Fibroblasts, Endothelial cells, Epithelial cells, Monocytes, Adipocytes, CD8+ T-cells. **(C, D)** Expression of the 5 characterized genes in the 6 cell categories.

Using the Seurat software, we conducted a single-cell analysis after downloading the single-cell data from the GSE181316 dataset. The tSNE technique was used to cluster the cells, producing 36 different isoforms (Figure 5A). The 36 clusters were divided into six cell types using the SingleR R package to annotate every subtype: fibroblasts, endothelial cells, epithelial cells, monocytes, adipocytes, and CD8^+^ T-cells (Figure 5B). Figures 5C, D shows the expression levels of important genes within each of these six cell types. According to our investigation, endothelial cells, epithelial cells, monocytes, and adipocytes are the main cell types that exhibit high expression of SNCA. Similarly, SLC38A1 exhibited high expression in endothelial cells, monocytes, adipocytes, epithelial cells, and CD8^+^ T-cells. SOCS2 was predominantly expressed in endothelial cells and adipocytes. AKR1C3 showed high expression in fibroblasts, endothelial cells, and adipocytes, while PLIN2 was highly expressed across fibroblasts, monocytes, endothelial cells, CD8^+^ T-cells, adipocytes, and epithelial cells.

### 3.4 Analysis of key gene infiltration by immune cells

**FIGURE 6:**
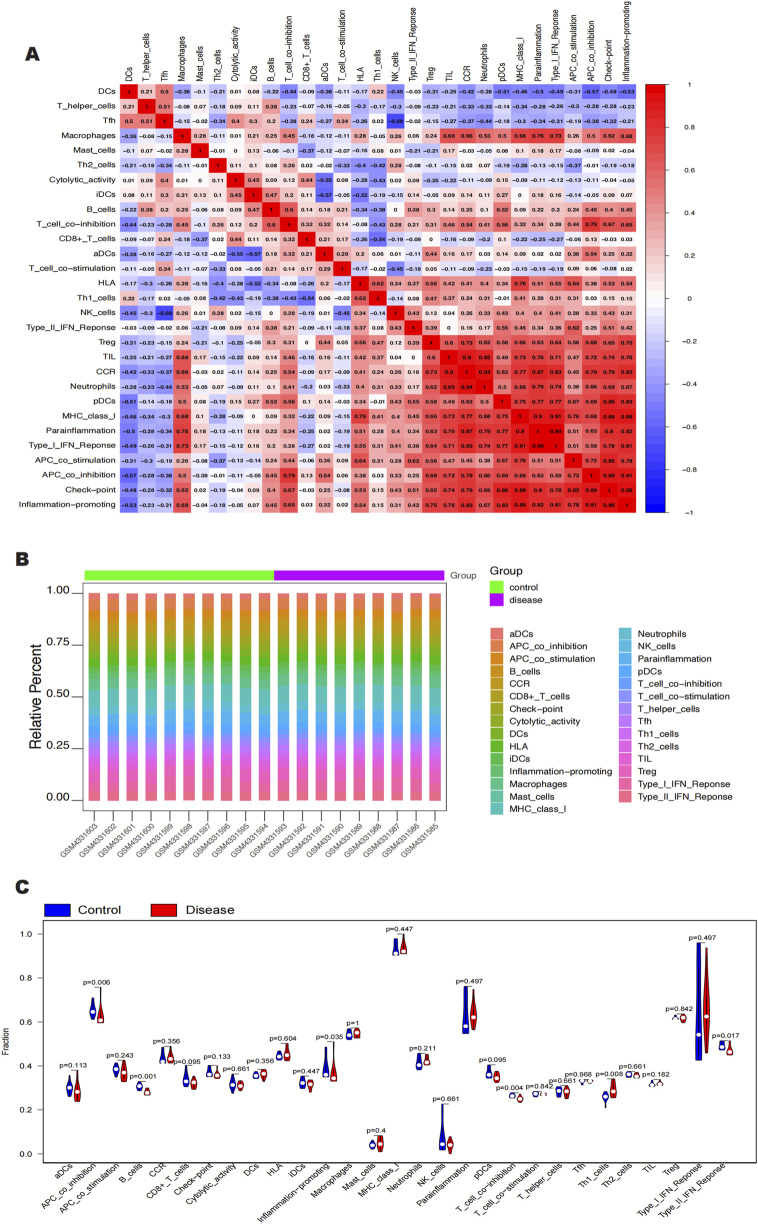
**(A, B)** Immune cell content as a percentage of each patient and correlation between immune cells. **(C)** APC_co_inhibition, B cells, Inflammation-promoting. T_cell_co_inhibition, Thl_cells, and Type_II_IFN_Reponse were significant in intergroup expression.

**FIGURE 7:**
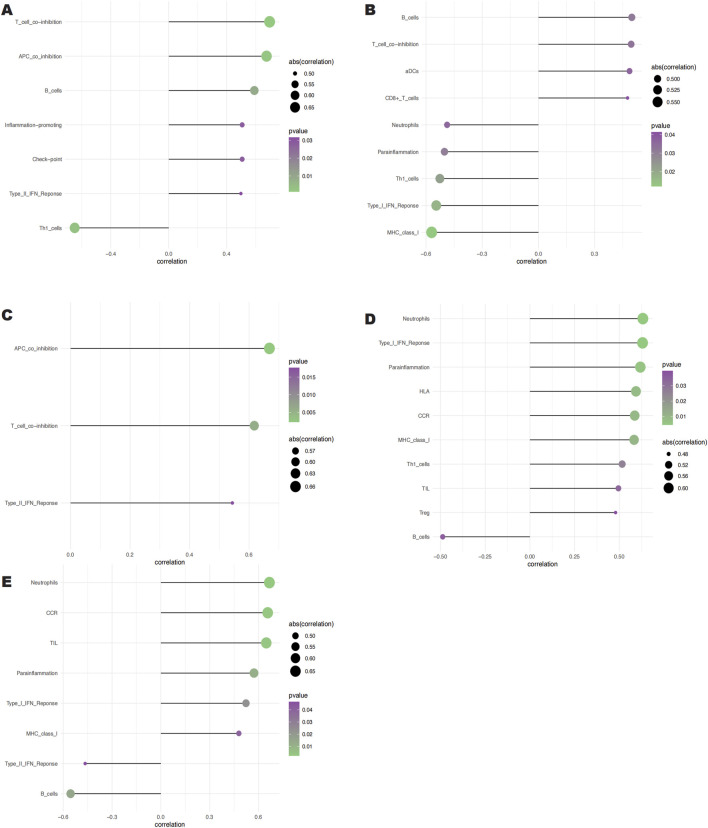
Relationships between the 5 key genes and immune cells. **(A)** Relationship between SNCA and immune cells. **(B)** Relationship between SLC38A1 and immune cells. **(C)** Relationship between SOCS2 and immune cells. **(D)** Rela- tionship between AKR1C3 and immune cells. **(E)** Relationship between PLIN2 and immune cells.

Clinical therapeutic response and disease diagnosis are significantly influenced by the intricate interplay of immune cells, growth factors, extracellular matrix components, inflammatory mediators, and specific physicochemical features that comprise the microenvironment. There is a notable association between fibrosis and immune infiltration, particularly during inflammatory diseases and tissue repair processes (Brandt et al., 2020; Distler et al., 2019; Rosenbloom et al., 2017). In order to get further insight into the connection between important genes and immune infiltration, we examined the keloid dataset to determine the possible biochemical pathways by which these genes affect the development of keloid formation. The figure (Figures 6A, B) shows the percentage of immune cells present in each patient and the connections between different immune cells. Notably, we found that the groups differed significantly in the expression of APC co-inhibition, B cells, molecules that promote inflammation, T cell co-inhibition, Th1 cells, and Type II IFN response (Figure 6C). We also looked at the connections between immune cells and key genes. According to our research, SNCA has a substantial negative correlation with Th1 cells but a significant positive correlation with T cell and APC co-inhibition (Figure 7A). SLC38A1 exhibited a positive association with Type II IFN response, a substantial negative correlation with MHC class I, and significant positive correlations with B cells and T cell co-inhibition (Figure 7B). Significant positive relationships were found between SOCS2 and the Type II IFN response, T cell co-inhibition, and APC co-inhibition (Figure 7C). Furthermore, AKR1C3 was considerably negatively connected with B cells and significantly positively correlated with neutrophils and the Type I IFN response (Figure 7D). PLIN2 also showed a substantial negative correlation with Type II IFN response and B cells, but a significant positive correlation with neutrophils and CCR (Figure 7E).

### 3.5 Key genes and various immune factor correlation

**FIGURE 8:**
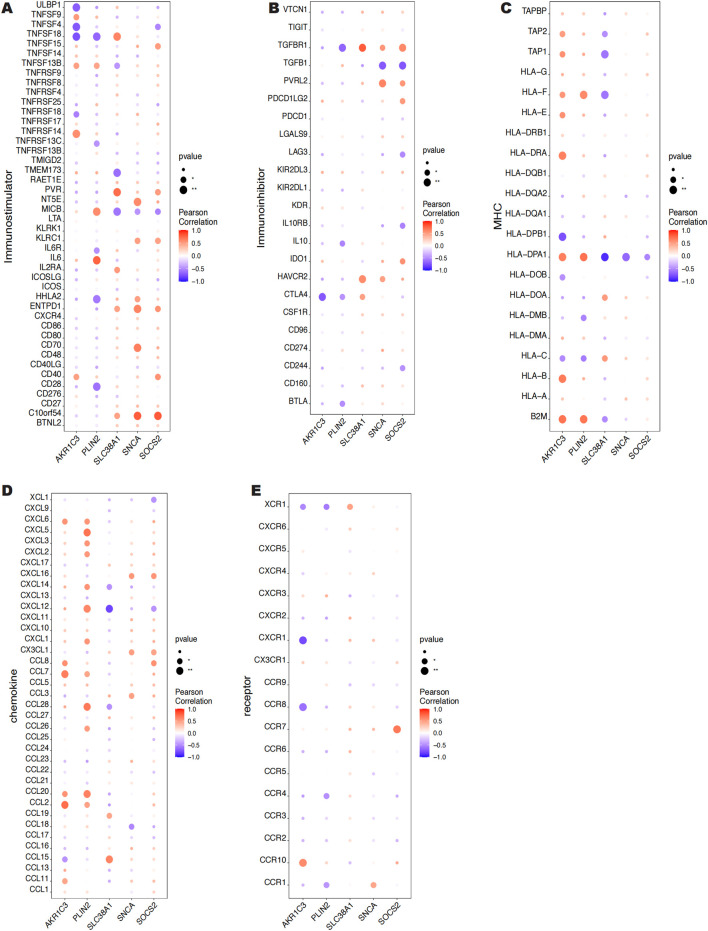
Correlation between 5 key genes and immunomodulatory factors, MHC, chemokines and cellular receptors.

Using information from the TISIDB database, We looked at the connections between five key genes and additional immunological elements, including immunomodulators (Figures 8A, B), MHC (Figure 8C), chemokines (Figure 8D), and cellular receptors (Figure 8E). Our results indicate that the key genes are essential to the immunological milieu and that the level of immune cell infiltration is highly linked with them.

### 3.6 Gene set enrichment analysis

**FIGURE 9:**
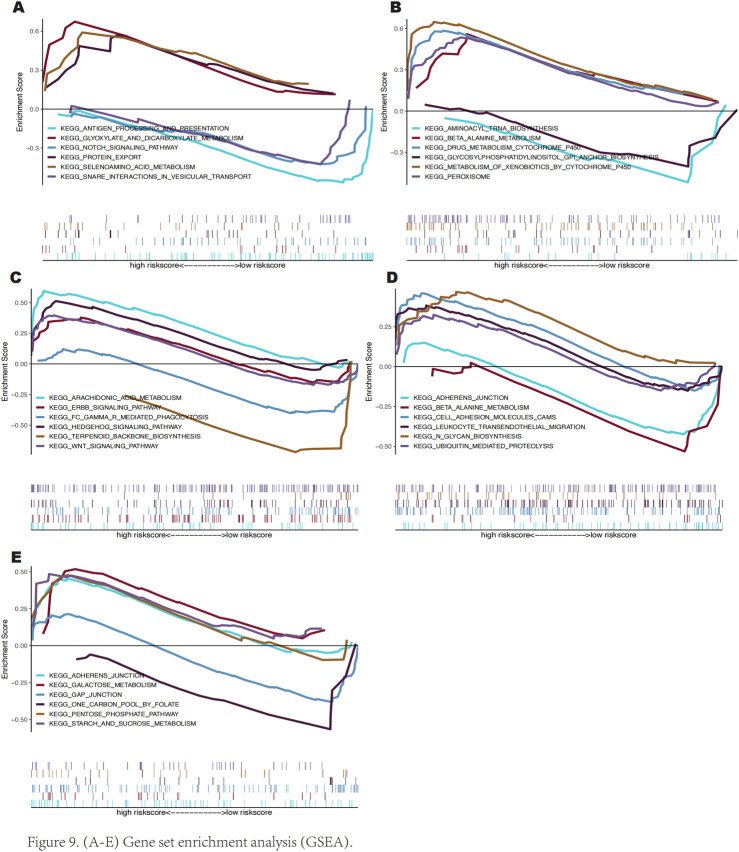
Gene pathway enrichment analysis (GSEA).

We used GSEA to investigate the possible molecular pathways via which these important genes affect keloid growth. The results revealed that SLC38A1 is rich in pathways like the presentation and processing of antigens, Notch signaling, and protein export (Figure 9A). AKR1C3 is enriched in peroxisome, drug metabolism via cytochrome P450, and beta-alanine metabolism pathways (Figure 9B). SOCS2 exhibits enrichment in the ErbB, Hedgehog, and Wnt signalling pathways (Figure 9C). PLIN2 is enriched in galactose metabolism, the pentose phosphate pathway, and gap junctions (Figure 9D). SNCA is enriched in adherens junctions, beta-alanine metabolism, and cell adhesion molecules (CAMs) pathways (Figure 9E).

### 3.7 Gene set variation analysis

**FIGURE 10:**
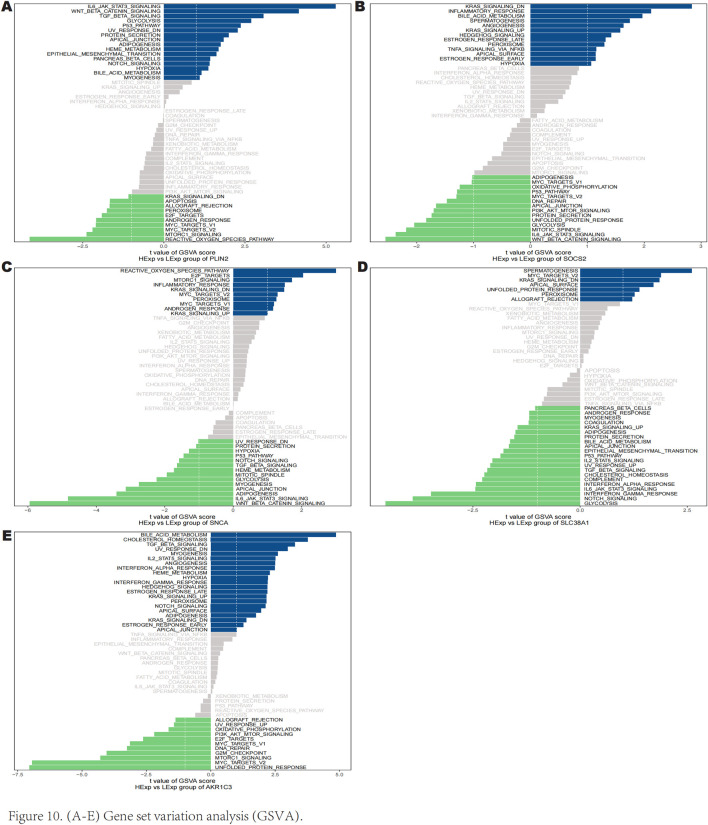
Gene set variance analysis (GSVA).

The GSVA results indicated that high expression of PLIN2 is enriched in several signaling pathways including IL-6 JAK-STAT3 signaling, Wnt/β-catenin signaling, p53 pathway, and TGF-β signaling (Figure 10A). Similarly, high expression of SOCS2 is predominantly associated with downregulated KRAS signaling, inflammatory response, hedgehog signaling, and peroxisome pathways (Figure 10B). High expression of SNCA was found to enrich the reactive oxygen species pathway, E2F targets, and mTORC1 signaling (Figure 10C). Furthermore, high expression of SLC38A1 is correlated with downregulated KRAS signaling, MYC targets v2, and peroxisome pathways (Figure 10D). High expression of AKR1C3 is involved in bile acid metabolism, IL-2 STAT5 signaling, notch signaling, and TGF-β signaling (Figure 10E). These findings suggest that key genes may influence the progression of keloids through these pathways.

### 3.8 Validation of key genes

**FIGURE 11:**
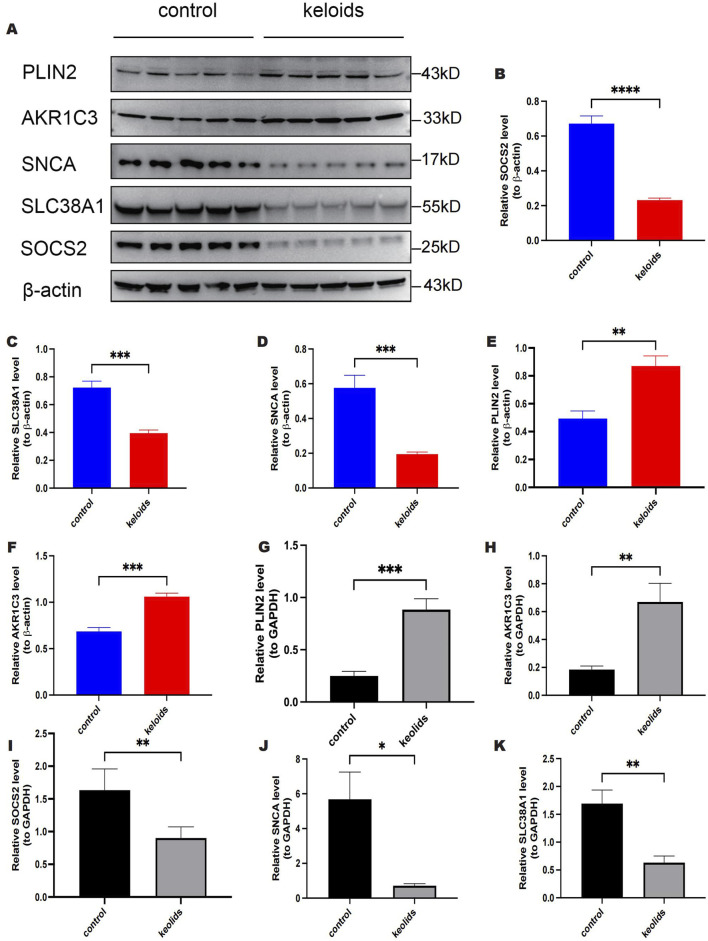
Expression of PLIN2, AKRIC3, SOCS2, SLC38A1 and SNCA in validation samples at protein and mRNA levels. **(A)** Protein blot analysis of PLIN2, AKRIC3, SOCS2, SLC38A1 and SNCA. **(B)** Relative quantitative results of Western Blot detection of SOCS2 expression. **(C)** Relative quantitative results of Western blot detection of SLC38A1 expression. **(D)** Relative quantitative results of Western blot detection of SNCA expression. **(E)** Relative quantitative results of PLIN2 expression detected by Western blot. **(F)** Relative quantitative results of AKRIC3 expression detected by Western blot. **(G)** RT-qPCR results of PLIN2. **(H)** RT-qPCR results of AKR1C3. **(I)** RT-qPCR results of SOCS2. **(J)** RT-qPCR results of SNCA. **(K)** RT-qPCR results of SLC38A1.

We further validated the expression of five key genes in scar keloid and normal skin samples using RT-qPCR and Western blot analyses. We examined mRNA levels of these genes in five normal and five keloid samples. As shown in (Figures 11A–F), at the protein level, the expression of PLIN2 and AKR1C3 was significantly increased in keloid samples compared to normal samples, whereas the expression of SOCS2, SLC38A1, and SNCA was significantly decreased. Additionally, as depicted in (Figures 11G–K), at the mRNA level, there was also a significant increase in the expression of PLIN2 and AKR1C3 in keloid samples, and a significant decrease in the expression of SOCS2, SLC38A1, and SNCA. These findings are consistent with the expression patterns of key genes identified during the screening phase.

## 4 Discussion

Keloids represent an abnormal formation of fibrous tissue, typically developing on injured skin. Excessive tissue formation often results from overgrowth of granulation tissue or increased Type III collagen during the healing process. To date, the pathogenesis of keloids remains elusive. These scars are notoriously difficult to treat due to their tendency to recur after excision and significantly affect patients’ quality of life, stemming from both aesthetic concerns and functional impairments that can lead to psychological distress. Therefore, identifying molecular characteristics that can effectively prevent progression and enhance the prognosis of keloids is crucial.

Ferroptosis is a distinct mode of cell death, regulated by various cellular metabolic processes. The accumulation of intracellular iron and lipid peroxidation are two critical signals for membrane oxidative damage during ferroptosis (Rochette et al., 2022). Excessive production of reactive oxygen species (ROS) within cells, beyond their scavenging capacity, leads to oxidative stress (Sies et al., 2022). The formation of keloid scars largely manifests oxidative stress, appearing to result from dysregulated wound healing. Excessive inflammation or local hypoxia can distort wound repair, generating an abundance of free radicals and ROS. Tissue mediators such as transforming growth factor-beta (TGF-β) can induce angiogenesis and lead to wound fibrosis (Distler et al., 2019; Lichtman et al., 2016; Muppala et al., 2017). Ferroptosis is linked to the pathobiology of organ fibrosis and may impact nearly all organ systems, including the skin, lungs, heart, liver, and kidneys (Liu and Wang, 2022; Wang et al., 2023). Therefore, we have investigated the potential mechanisms by which ferroptosis influences the development of keloids.

Firstly, we used GEO database data mining to get transcriptome data from both normal and keloid skin tissue. By creating difference sets from the control and disease-related DEGs and crossing them with ferroptosis genes, we were able to identify 14 hub genes linked to keloid formation. According to the GO functional enrichment study, the functional module’s genes were connected to monocarboxylic acid metabolic processes, carboxylic acid transport, pathways of neurodegeneration in multiple diseases, epithelial cell differentiation, and molecular adaptor activity. According to reports, energy metabolism and cell signaling are involved in the metabolism of monocarboxylic acid, and these procedures could be essential for the development, spread, and penetration of tumor cells (Halestrap and Wilson, 2012; Payen et al., 2020). It has been demonstrated that the pathway of neurodegeneration in multiple diseases is involved in oxidative stress and inflammatory responses (Michael et al., 2019). The epithelial cell differentiation pathway directly impacts the reconstruction of the skin epidermis and repair processes (Caldon et al., 2010). These mechanisms may occur in keloids. Research has shown that oxidative stress is linked to keloids, and that apoptosis, cell proliferation, and inflammatory responses may have an impact on keloids’ development.

The LASSO regression reduced the dimensionality of the feature module from 14 genes to five key genes, revealing upregulation of AKR1C3 and PLIN2, and downregulation of SOCS2, SLC38A1, and SNCA in the initial sequencing results. Subsequently, the accuracy of the model’s predictions was investigated using ROC curves, and the diagnostic efficacy of AKR1C3, PLIN2, SOCS2, SLC38A1, and SNCA was validated through an external dataset. Single-cell sequencing analysis of the five genes annotated them to certain cell types significantly associated with disease pathogenesis. We employed ssGSEA to calculate and compare the proportions of different immune cells within scar keloid samples. This approach allowed us to assess the relationships between key genes and various immune cells, as well as to explore the correlations between these genes and different immune factors. Additionally, results from GSEA and GSVA provided evidence of the potential pathways and functions involving AKR1C3, PLIN2, SOCS2, SLC38A1, and SNCA. Finally, the expression of these genes was validated using qRT-PCR and Western blot analyses.

We conducted further research on the five key genes identified through model selection. These five genes are predominantly expressed in six cell types: fibroblasts, endothelial cells, epithelial cells, monocytes, adipocytes, and CD8^+^ T-cells. Fibroblasts play a crucial role in scar formation by synthesizing and secreting extracellular matrix components, such as collagen, thereby facilitating tissue remodeling during wound healing. Studies have shown that excessive accumulation of iron leads to increased lipid peroxidation, which damages cell membranes and subsequently induces apoptosis in fibroblasts (Hu et al., 2024). Ferroptosis may indirectly influence scar formation through multiple mechanisms, including the induction of fibroblast apoptosis, modulation of their function, regulation of inflammatory responses, and alterations in iron metabolism (Yin et al., 2024). Endothelial cells are vital for angiogenesis and the maintenance of vascular permeability. During the scar healing process, they support the repair of surrounding tissues by providing oxygen and nutrients. Research indicates that ferroptosis induces endothelial cell damage and dysfunction, increases oxidative stress and inflammatory responses, and inhibits angiogenesis (Yu et al., 2021). Damage to endothelial cells results in localized hypoxia, which further impacts the function of fibroblasts, promoting their transition towards fibrosis, a process that contributes to the formation and progression of scars (Bai et al., 2020; Raslan et al., 2024). Epithelial cells are essential for the barrier function of the skin and other organs and primarily participate in tissue regeneration and repair. Luo et al. found that ferroptosis in epithelial cells may affect their proliferation and migration capabilities, thereby influencing wound healing and scar formation (Luo et al., 2024). Another study confirmed that ferroptosis induces oxidative stress and lipid peroxidation, leading to the death and dysfunction of keratinocytes (Vats et al., 2021). Consequently, this epithelial cell damage increases inflammatory responses and promotes fibroblast activation, which further enhances scar formation (Deng et al., 2023). Monocytes play a significant role in inflammatory responses and immune responses, differentiating into macrophages that participate in tissue repair. Research has demonstrated that ferroptosis contributes to lung fibrosis by driving macrophage polarization, fibroblast proliferation, and extracellular matrix deposition (Hu et al., 2024). These processes may also relate to scar formation. Ferroptosis induces M1 macrophage polarization, enhancing their role in inflammatory responses and resulting in the release of additional pro-inflammatory factors (Handa et al., 2019; Zhou et al., 2018), thereby promoting fibroblast proliferation and collagen synthesis, which exacerbates scar formation. Adipocytes are crucial for energy storage and metabolic regulation and are closely associated with inflammatory responses (Lindhorst et al., 2021). Iron acts as an important regulator in the energy metabolism of adipose tissue (Ma et al., 2021). The occurrence of ferroptosis is often associated with oxidative stress. Under oxidative stress conditions, adipocytes may release inflammatory factors, leading to inflammation in surrounding tissues. The formation of keloids is closely linked to chronic inflammation; thus, the regulation of ferroptosis in adipocytes may indirectly influence the occurrence of keloids. CD8^+^ T cells play a vital role in defending against viral and bacterial infections and in tumor immunity (Pritykin et al., 2021). However, their role in scar formation remains unclear. Studies have found that ferroptosis in CD8^+^ T cells can weaken their anti-tumor and anti-infection capabilities (Li et al., 2024). Imbalances in the immune microenvironment promote inflammatory responses, which may further correlate with scar formation. Single-cell sequencing results indicate that ferroptosis-related genes expressed in the aforementioned cell types primarily participate in processes such as lipid metabolism, oxidative stress, and inflammatory responses, all of which may significantly influence fibrosis and scar formation.

The results of immune infiltration analysis reveal that these five genes exhibit significant correlations with various immune cell types, including antigen-presenting cell (APC) co-inhibition, B cells, inflammation-promoting cells, T cell co-inhibition, Th1 cells, and the Type II interferon response. These immune cells not only effectively respond to infections and tumors but also maintain the immune balance of the organism by regulating immune responses (Chen and Flies, 2013; Krieg et al., 2007; Wynn, 2015; Zeng et al., 2018; Zhang et al., 2015). Specifically, SNCA shows a significant positive correlation with T cell co-inhibition and APC co-inhibition, suggesting its potential role in regulating immune responses. In contrast, its significant negative correlation with Th1 cells may indicate that SNCA plays a role in suppressing certain types of immune responses. SLC38A1 is significantly positively correlated with both B cell and T cell co-inhibition, indicating its involvement in modulating the activity of these cells. Additionally, its significant negative correlation with the Type II interferon response and MHC Class I molecules suggests that SLC38A1 may exert an inhibitory effect on antigen presentation and cellular immune responses. Furthermore, SOCS2 is significantly positively correlated with APC co-inhibition, T cell co-inhibition, and the Type II interferon response, highlighting its crucial role in regulating immune cell activity and inflammatory responses (Yoshimura et al., 2007). AKR1C3 shows a significant positive correlation with neutrophils and the Type I interferon response, which may indicate its role in promoting inflammatory responses. Its significant negative correlation with B cells suggests that AKR1C3 may inhibit B cell activity. Moreover, PLIN2 is significantly positively correlated with neutrophils and CCR, indicating its potential role in regulating immune cell infiltration and inflammatory responses (Reiss et al., 2001). The significant negative correlation with B cells and the Type II interferon response suggests that PLIN2 may have an inhibitory effect on B cell activity and immune responses. Additionally, the five genes exhibit significant correlations with various immune factors, including immunostimulators, MHC molecules, receptors, immunoinhibitors, and chemokines. These molecules interact through intercellular signaling and regulation, determining the intensity and type of immune system responses (Alberto et al., 2004; Dhatchinamoorthy et al., 2021; Deng et al., 2022). The aforementioned findings further confirm the close relationship between the expression of these five ferroptosis-related genes and the level of immune cell infiltration. It is well-established that activated immune cells, such as macrophages and neutrophils, generate substantial amounts of ROS. Continuous oxidative stress may activate various inflammatory signaling pathways, such as NF-κB and JAK-STAT, which typically exacerbate inflammatory responses (Banerjee et al., 2017; Lawrence, 2009). Prolonged inflammation can stimulate the activation and proliferation of fibroblasts, promoting the occurrence of fibrosis, and may play a critical role in the formation of keloids.

Previous studies have found that PLIN2 is associated with the perilipin protein family and plays a major role in regulating intracellular lipid metabolism and the formation of lipid droplets (Kimmel and Sztalryd, 2016). In hepatocellular carcinoma (HCC), overexpression of PLIN2 promotes cell proliferation, potentially by inhibiting the degradation of HIF1α, which facilitates HCC cell proliferation (Liu et al., 2022). Another study indicates that PLIN2 deficiency reduces triglyceride (TG) levels in wild-type mouse embryonic fibroblasts (MEFs) by enhancing autophagy and can prevent fatty liver disease (Tsai et al., 2017). The upregulation of PLIN2 in keloids may influence the scar healing process by promoting cell proliferation and reducing autophagy. AKR1C3 plays a crucial role in androgen and steroid metabolism and is highly expressed in metastatic prostate cancer specimens and circulating tumor cells (Penning, 2019). AKR1C3 is significantly upregulated in HCC and promotes tumor proliferation and metastasis through the activation of NF-κB signaling (Wu et al., 2022; Zhou et al., 2021). The upregulation of AKR1C3 in keloids may contribute to scar formation by affecting cell proliferation and extracellular matrix component deposition. SOCS2, a negative regulatory factor, primarily modulates cell growth and differentiation by inhibiting cytokine signaling pathways, and its abnormal regulation is associated with various inflammatory and cancerous diseases (Rico-Bautista et al., 2006). Research shows that SOCS2 can enhance IL-2 and IL-3-induced STAT phosphorylation and promote cytokine-induced cell proliferation (Tannahill et al., 2005). High SOCS2 expression facilitates the ubiquitination and degradation of SLC7A11, promoting ferroptosis in HCC. Targeting SOCS2 may improve the efficiency of radiotherapy and patient prognosis in HCC (Chen et al., 2023). In keloids, the expression of SOCS2 is downregulated, which may affect the scar healing process by modulating inflammation, cell proliferation, and other pathways. SLC38A1 encodes an amino acid transporter involved in regulating amino acid metabolism both intracellularly and extracellularly (Kandasamy et al., 2018; Schiöth et al., 2013). Studies have shown that knockout of SLC38A1 significantly inhibits HCC cell growth and migration, and that SLC38A1 regulates the occurrence and progression of HCC through glutamine-mediated energy metabolism by modulating the PI3K/AKT/mTOR signaling pathway (Hg et al., 2023). In research on the pathogenesis of selective intrauterine growth restriction (sIUGR), overexpression of hypoxia-inducible factor (HIF-1α) induces upregulation of SLC38A1, which reduces cell growth and migration (Chen et al., 2022). In bleomycin (BLM)-induced pulmonary fibrosis rat lung tissues and transforming growth factor-β1-treated HFL1 cell models, downregulation of miR-150-5p targets SLC38A1 expression, leading to increased expression of inflammatory cytokines, fibroblast activation, and elevated ROS levels (Yang et al., 2020). Our research finds that SLC38A1 is downregulated in keloids, suggesting that it may have a significant impact on processes such as inflammation and cell proliferation. SNCA encodes α-synuclein, which is widely expressed in the nervous system and is associated with neuron survival, synaptic function, and cellular stress responses (Burré et al., 2018). Numerous studies indicate that SNCA is linked to tumorigenesis; its deficiency disrupts iron metabolism, leading to ferritin-iron accumulation and cell apoptosis (Shekoohi et al., 2021). Overexpression of SNCA in medulloblastoma can inhibit tumor invasion and induce apoptosis (Li et al., 2018). And SNCA overexpression may inhibit the proliferation of lung adenocarcinoma cells via the PI3K/AKT pathway (Zhang et al., 2022). The differential expression of SNCA in keloids may influence scar formation through processes such as cytokine regulation, cytokine signaling, iron metabolism, and neuroregulation. In summary, the upregulation of AKR1C3 and PLIN2 in keloids may inhibit ferroptosis by affecting oxidative stress, as well as lipid metabolism and storage, thereby promoting cell proliferation and fibrosis, which further contributes to scar tissue formation. Additionally, changes in the expression of SOCS2, SLC38A1, and SNCA are closely related to the regulation of ferroptosis by oxidative stress, inflammatory responses, and imbalances in iron metabolism. The interactions among these processes may significantly impact cell proliferation, apoptosis, and fibrosis, collectively promoting the formation of keloids.

GSEA and GSVA results indicate that high expression of SNCA is enriched in the reactive oxygen species pathway, E2F targets, and MTORC1 signaling, which play roles in cell growth regulation, redox balance, and cellular metabolic stress responses (Kent and Leone, 2019; Napolitano et al., 2022; Thannickal and Fanburg, 2000). High expression of SLC38A1 is enriched in KRAS signaling dn, MYC targets v2, and Peroxisome pathways, primarily regulating cell proliferation, growth, metabolic energy balance, and redox status (Bryant et al., 2014; Okumoto et al., 2020; Schulze et al., 2020). High expression of AKR1C3 is enriched in bile acid metabolism, IL2 STAT5 signaling, NOTCH signaling, and TGF-β signaling, which are involved in cell proliferation and growth regulation, metabolic and energy balance, immune regulation, and inflammatory responses (Chiang, 2015; Distler et al., 2019; Meurette and Mehlen, 2018; Ross and Cantrell, 2018). Similarly, high SOCS2 expression is enriched in KRAS signaling dn, Hedgehog signaling, inflammatory response, and Peroxisome pathways, participating in cell metabolism regulation, proliferation, growth control, and redox balance (Bryant et al., 2014; Ingham, 2022; Okumoto et al., 2020). Moreover, high PLIN2 expression is enriched in IL6 JAK STAT3 signaling, WNT/β-catenin signaling, P53 Pathway, and TGF-β signaling, which interact in cell proliferation, growth regulation, inflammatory response, immune modulation, and tissue regeneration (Clevers and Nusse, 2012; Joerger and Fersht, 2016; Zegeye et al., 2018). The results indicate that the pathways enriched by these five genes are not only involved in cell proliferation and growth but also interact with processes of oxidative stress and inflammatory responses. Ferroptosis is widely recognized for promoting lipid peroxidation and oxidative stress, leading to cell death and inflammation, thereby accelerating the fibrotic process. These processes play a crucial role in the regulation of ferroptosis and may have a significant impact on the formation of keloids.

In summary, we identified AKR1C3, PLIN2, SOCS2, SLC38A1, and SNCA as characteristic genes associated with ferroptosis in keloids through comprehensive transcriptomic data analysis. Additionally, we collected and validated samples, confirming these genes as molecular markers of ferroptosis in keloids and further exploring the role of ferroptosis in the fibrotic process. However, this study has certain limitations, primarily regarding the reliance on transcriptomic data and the urgent need for functional validation. While transcriptomic analysis can reveal gene expression changes in specific samples, it may not fully reflect cellular behaviors across different individuals or pathological states. Although some genes exhibit significant differences in transcriptomic analysis, their functions in cellular or animal models have not been adequately validated. To address these limitations, future research will focus on systematic functional validation of the key genes identified in the transcriptomic analysis. This will include experiments such as gene knockout, overexpression, and small molecule inhibition to observe the effects of these genes on fibroblast survival, proliferation, and collagen synthesis. Through these studies, we aim to achieve a more comprehensive understanding of the specific mechanisms linking ferroptosis to keloid formation. This approach provides new insights for regulating cellular death mechanisms by targeting ferroptosis-related genes, potentially paving the way for developing novel therapeutic strategies.

## Data Availability

The datasets presented in this study can be found in online repositories. The names of the repository/repositories and accession number(s) can be found in the article/supplementary material.

## Abbreviations

DEGs, Differentially expressed genes; GEO, Gene Expression Omnibus; LASSO, Least absolute shrinkage and selection operator; AUC, Area under the receiver operating characteristic curve; GO, Gene ontology; ROC, Receiver operating characteristic curve; GSVA, Gene Set Variation Analysis; GSEA, Gene set enrichment analysis; BCA, Bicinchoninic Acid; RIPA, Radioimmunoprecipitation Assay.
